# North Korea’s COVID-19 policy dilemma: epidemic prevention conflicting with trade

**DOI:** 10.1186/s12992-023-01013-9

**Published:** 2024-01-16

**Authors:** Byungjin Park, Joonmo Cho

**Affiliations:** 1 HRD Center and Department of Economics of SKKU, Sungkyunkwan University, 25-2, Sungkyunkwan-ro, Jongno-gu, Seoul, 03063 Korea; 2 Department of Economics in SKKU, Sungkyunkwan University, 25-2, Sungkyunkwan-ro, Jongno-gu, Seoul, 03063 Korea

**Keywords:** Border closure, COVID-19 prevention, North Korea, Trade

## Abstract

**Background:**

Amidst the COVID-19 pandemic, striking a delicate balance between sustaining economic activity and safeguarding public health has become a crucial concern. The border closures for COVID-19 prevention have further intensified concerns for North Korea, which conducts over 90% of its trade with China and Russia, countries sharing its borders.

**Methods:**

This study aims to scrutinize North Korea’s response to these competing imperatives by examining the impact of border closures on the country’s trade dynamics with China and Russia. This study employed the difference-in-difference (DID) method to analyze transformations in North Korea’s economic activity before and after the border closures, utilizing trade data and nighttime satellite imagery captured in 2019 and 2020.

**Results:**

The results reveal that North Korea actively reoriented its trade priorities towards Russia and accorded precedence to its epidemic prevention system over its economy during the pandemic. A noticeable increase in imports of food and pharmaceuticals was observed, indicating a significant rise in the inflow of these essential commodities.

**Conclusion:**

The findings of this study suggest that during the early stages of the COVID-19 pandemic, North Korea placed significant policy emphasis on preserving public health. However, due to economic hardships and food shortages, if the pandemic persists in the long term, it indicates the possibility of partial or complete lifting of border closures to mitigate these challenges.

## Background

Since the onset of the COVID-19 pandemic in 2020, nations worldwide have implemented various regulations to curb the spread of infection. Initially, policies prioritizing public health measures over economic considerations were enforced, with mobility restrictions being a prominent component [1–5]. However, these measures posed a dilemma, forcing a choice between public health and economic priorities [6, 7]. The mobility restrictions play a positive role in preserving public health, but they hinder economic activities by regulating people’s movements. Accordingly, as the situation gradually improved and vaccines became more widely available, the utilization of such policies diminished. Furthermore, in the pursuit of economic recovery, policies centered around disease control have been flexibly applied in accordance with evolving circumstances.

Differing from the approaches taken by many countries in response to the COVID-19 pandemic, North Korea, a closed economic system, has implemented border closures since 2020 to prevent the entry of the coronavirus. Examining North Korea’s actions in conjunction with its socio-economic conditions reveals a distinct situation. While other nations face a dilemma of choosing between public health and the economy, North Korea appears to be in a situation where it could potentially lose both aspects by opting for a closed economic system and border closure to combat the virus. North Korea ranks 198th out of 212 countries in terms of GDP per capita and is a country with limited economic freedom [8]. North Korea relies on China and Russia for over 90% of its trade, and there is almost no trade activity with other countries [9]. Furthermore, owing to the repeated nuclear development and testing, economic sanctions by the UN have become increasingly severe, leading to a predicted negative growth rate for the North Korean economy in both 2017 and 2018 [10]. Additionally, the United States has imposed its own economic sanctions on North Korea, along with the UN [11]. On account of these factors, the trade disruption caused by border closures means a serious deterioration in North Korea’s national economy, and in particular, the country’s chronic food shortages may be accelerated by epidemic prevention policies, worsening the health of its citizens. Accordingly, North Korea is in a situation where it needs to carefully implement epidemic prevention policies while considering its economic situation, which is a distinct difference from other countries [12–14].

North Korea, located along the border with China, was in a vulnerable position, facing significant repercussions if the coronavirus were to infiltrate the country. In terms of healthcare, the collapsed medical system since the famine compelled North Korean residents to individually procure medical supplies from informal markets. Due to the outdated healthcare facilities and environment, obtaining medical services was practically impossible [15, 16]. Economically, continuous nuclear tests subjected North Korea’s trade activities to the impact of UN economic sanctions. With a high dependence on imports from China, the situation posed a risk of a national-level economic shock if trade with China became challenging. Additionally, chronic food insecurity affected 41.6% of the entire population in 2019, resulting in nutritional deficiencies, particularly among children, with persistent stunting [17]. In such circumstances, the socio-economic impact of a virus incursion represented a threat at the national level for North Korea, resembling a choice between survival and demise.

This study aimed to analyze North Korea’s decision-making process regarding the contrasting policy choices of economy and epidemic prevention amidst the challenges posed by the COVID-19 pandemic. Obtaining official data from the country is a major challenge in researching North Korea. To supplement this issue, the study utilized trade data stored in China, which has the highest trade volume with North Korea, and information provided by the UN Comtrade and South Korea. To ensure objectivity, the study also compared changes in North Korea using NASA’s night-time light intensity data as an additional source of information, rather than relying solely on unofficial sources. Overall, this study sheds light on the trade and economic situation of North Korea, and provides valuable insights into the country’s decision-making process in the face of the complex challenges posed by the pandemic.

This study analyzes North Korea’s decision-making process regarding economy and epidemic prevention by examining the impact of COVID-19 border closures on the country’s economy. The study addresses three main questions: (1) To what extent did the border closures disrupt North Korea’s trade activities? (2) Did COVID-19 result in differences in North Korea’s trade with China and Russia? (3) Which area did North Korea prioritize in its policy choices between economy and epidemic prevention?

### North Korea’s COVID-19 response

**Fig. 1:**
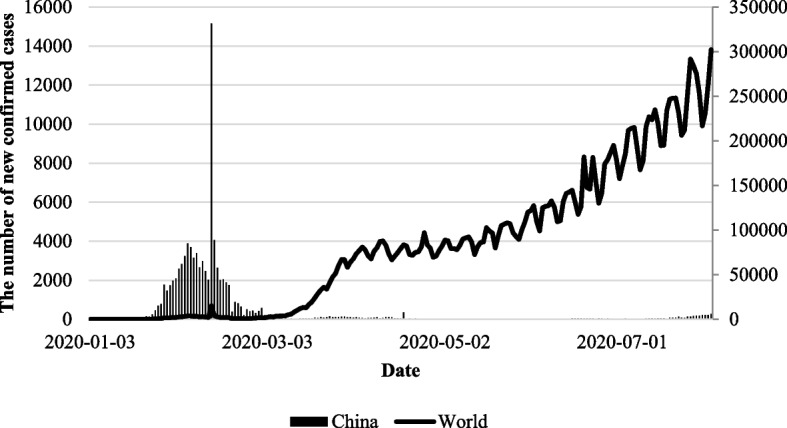
Trends in the number of new COVID-19 cases in China and worldwide. Note: The bar and line graphs represent trends in the number of new confirmed COVID-19 cases in China and worldwide, respectively. The left y-axis shows the number of confirmed cases in China, and the right y-axis shows the number of confirmed cases worldwide. The above data are published by WHO [21]

North Korea has taken the strongest response externally to COVID-19. When the spread of the coronavirus was accelerating in China, which borders North Korea, North Korea pre-emptively closed its border [18]. Figure 1 shows the trend of newly confirmed cases in China and worldwide. Since January 22, travelers entering North Korea have been limited. On January 23, a national emergency was declared and preparations to prevent the spread of COVID-19 were initiated. On January 28, it closed all borders and blocked all international entries [12, 13]. Immediately after the closure, all foreigners who had symptoms similar to COVID-19 were isolated, and the entry and exit of travelers were completely cut off [19]. Furthermore, North Korea also suspended the operation of commercial airplanes for trade purposes in order to completely eliminate the possibility of the virus being introduced [20]. North Korea has maintained a closed system of national governance and limited diplomatic exchanges, resulting in its isolation from the international community. The border closure policy implemented for disease control has further exacerbated North Korea’s isolation.

Internally, North Korea has focused on efforts to prevent and control the spread of COVID-19 to avoid a collapse of its healthcare system. The country enforced the highest level of national emergency quarantine system and imposed strict restrictions on the movement of its citizens, while emphasizing the absolute obedience of the population to establish a robust quarantine system [12, 13]. Information on the epidemiological situation and measures for infection prevention were disseminated through the media to raise awareness of the disease [22]. The media coverage mainly included information on the number of cases, regional quarantine activities, knowledge on infectious diseases, and personal hygiene practices. Additionally, North Korea issued instructions to establish hospitals to address the vulnerable conditions of isolation facilities [23]. Other countries speculated that the coronavirus had already spread in North Korea, and due to the lack of adequate medical facilities, international assistance would be needed. However, North Korea officially announced that no confirmed cases of the coronavirus had been discovered and is maintaining a border lockdown [24].

## Methods

### Data

#### Night-time light

The utilization of satellite data is expanding to observe social and economic changes. In combination with artificial intelligence or machine learning, satellite data are used to observe and analyze changes in countries’ and communities’ socioeconomic conditions [25, 26]. Such data was recently used in the Russo-Ukrainian War to observe the transport of war equipment, the location of tanks, and the movement of combat units [27]. In particular, night-time light (NTL) satellite data are becoming increasingly useful for demonstrating economic progress, external economic shocks, and economic activities [28–32]. Compared to other data on North Korea, the changes in night-time light intensity captured by satellite imagery provide a more objective indicator of the country’s economic activity.

NTL data refers to data generated by the Visible Infrared Imaging Radiometer Suite Day-Night Band aboard the Suomi-NPP satellite of NASA and the National Oceanic and Atmospheric Administration [33]. The Earth Observation Group(EOG) provides monthly and yearly night illumination light images taken by satellites, and the image resolution is 15 arc seconds (approximately 500 m). There are two types of monthly files provided by EOG, vcm, which is a file that deletes the part affected by stray light, and vcmsl, which is a file that corrects it. In this study, we aimed to utilize as much information as possible by using vcmsl files that were corrected for stray light artifacts. In the GeoTiff file, avg_rade9h, which represents light information, is used; the unit of light is nW/cm^2/sr. GIS software was used to extract the light information from the downloaded light image files.

**Fig. 2:**
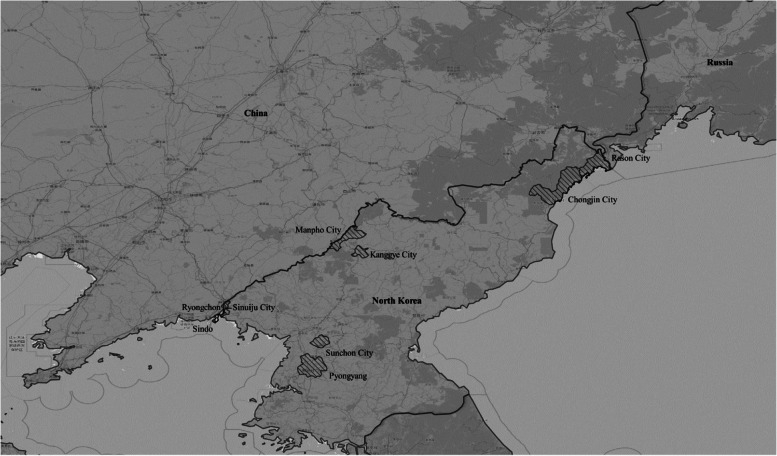
Location of North Korea-China and North Korea-Russia border trade cities. Note: The above figure is shown using the boundary map of North Korea’s administrative districts provided by the UN, Open Street Map, and World Map, and built into QGIS. The regional boundary map of North Korea utilized a file from the United Nations Office for the Coordination of Humanitarian Affairs (UNOCHA), which can be accessed through the link https://data.humdata.org/dataset/cod-ab-prk

We aimed to observe the changes in North Korea-Russia and North Korea-China trade after the border blockade; data were organized around trade cities located in the border area. Chongjin City and Rason City are North Korea-Russia regions, and Sindo, Sinuiju City, Ryongchon, Manpho City, and Kanggye City are North Korea-China regions. Figure 2 shows the location of each region. Kanggye, while not situated at the border between North Korea and China, serves as a hub for trade with China through its railway connection to Manpo. Similarly, Chongjin, not located at the border between North Korea and Russia, is recognized as a significant trade city, not only for its land-based trade connections through Rason City, but also for its potential for maritime trade. Thus, these cities were categorized and included in the analysis based on the group division.

An analysis was conducted targeting locations wherein lights appeared before border closure. In North Korea, electric energy use is sparse; only approximately 26% of the total population has access to electricity [34]. In addition, the aging and inefficient production methods of plants that produce electricity worsens the situation [35]. In this regard, we assumed that the locations of the light observed in North Korea’s trading cities in 2019 are where light is essential; thus, by analyzing the changes in light in these locations, we analyzed the change in trade activities after the border closure. The area where lights occurred was filtered using the 2019 lit mask v2.1, provided by the EOG [36]. This mask file provides the location of cells where light occurred in 2019; therefore, data in cells in areas with no light were treated as zero values. Table 1 shows the number of cells in each area and the statistics of the light intensity.

**Table 1:** Descriptive statistic of night-time light intensity by region

Location	N	Mean	SD
Chongjin City	5,530	0.781	0.794
Kanggye City	1,870	0.559	0.697
Manpho City	3,610	1.319	3.089
Pyongyang	24,940	1.765	3.458
Rason City	3,690	0.736	0.902
Ryongchon	1,950	1.596	3.733
Sindo	1,450	0.8	0.561
Sinuiju City	5,840	2.765	3.579
Sunchon City	5,090	0.683	0.815
Total	53,970		

The descriptive statistics are in cell units. The unit of light is nW/cm^2/sr

After the masking process using the lit mask file in 2019, 5,397 cells with light were identified, and 53,970 cells were used, corresponding to 10 months of data collection (October 2019 to July 2020). The number of cells was highest in Pyongyang (2,494), followed by Sinuiju (584) and Chongjin (553).

Compared with other cities, Pyongyang and Sinuiju had higher light intensities. This seems to be influenced by the cities’ roles. Sinuiju is a trading city where transactions between North Korea and China are the most active; it is an area with a transportation and logistics system that can deliver goods to other regions [37]. As Pyongyang is the capital of North Korea, where the country’s key government agencies are located, the lights appear strong.

#### Trade

**Fig. 3:**
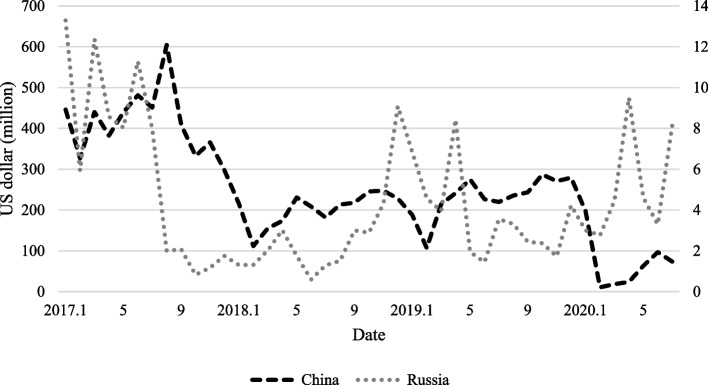
North Korea’s trade value with China and Russia. Note: The graph above was created using data from the General Administration of the Customs People’s Republic of China, UN Comtrade, and KITA

As North Korea does not have official data on economic indicators, it is necessary to compare and analyze various data from other sources. Accordingly, we explained the changes post-border closure through a comparative analysis involving trade data and the changes in the lights of trade cities in North Korea. We used trade data between North Korea and China disclosed by the General Administration of the Customs People’s Republic of China [38]. Data provided by the UN Comtrade and the Korea International Trade Association (KITA) were used as trade data between North Korea and Russia. Figure 3 shows the change in trade value between North Korea-China and North Korea-Russia.

North Korea’s trade continued to deteriorate in 2016 and 2017 as the UN’s economic sanctions tightened [39]. Although trade has gradually recovered since 2018, it experienced a sharp drop when border closure was implemented. Subsequently, North Korea’s trade with China decreased significantly, while its trade with Russia increased. Although the trade value increased as the number of confirmed COVID-19 cases in China decreased, it was considerably lower than the trade value before the border closure.

### Model

After the declaration of the COVID-19 pandemic, North Korea recommended restricting the movement of residents nationwide and implemented border closures. In other words, overall economic activities within North Korea are being influenced by orders for movement control. This study aims to observe changes in North Korea’s trade activities resulting from the border closure. To observe the additional impact on specific regions due to the border closure, beyond changes in economic activities resulting from movement control, a comparative analysis method between regions is necessary. To analyze these additional impacts, the assumption is required that the effects of movement control are uniformly felt across all regions of North Korea. Given the absolute authority and strong enforcement of orders by North Korea’s leadership during the COVID-19 pandemic, this study deems the application of such an assumption appropriate. Therefore, in this study, the difference-in-differences (DID) is used for regional comparative analysis to analyze the impact of the border closure on economic activities in the border regions.

To confirm the changes in the movements of North Korean trade cities located along the border after the border closure, we compared the changes in night-time lights before and after the official border closure declaration by the North Korean government. On January 22, 2020, North Korea closed the border; therefore, we chose January 2020 as the start of the border closure period. STATA 17 was used for analysis [40]. The light change was estimated using the following difference-in-difference (DID) regression model.

$${y}_{i,g,t}={\beta }_{0}+\sum\nolimits_{g\ne s}\sum\nolimits_{t\ne 4}{\beta }_{g,t}{Area}_{g}{Month}_{t}+{\delta }_{t}+{\theta }_{i}+{\varepsilon }_{i,g,t},$$

where ${y}_{i,g,t}$ represents logarithm values after adding 1 to the light value at cell $i$, in area $g$, and month $t$. As there are many values less than 1 in the light intensity of the cell unit, taking the logarithm directly of the light intensity of the cell unit results in many negative values. Therefore, 1 was added to the light value and logarithm was taken. $Area$ is a dummy variable that takes the value of 1 if the cell is in the corresponding region group and 0 otherwise; it is divided into four groups: North Korea-China Trade City, North Korea-Russia Trade City, Pyongyang, and Sunchon City. $Month$ is a dummy variable that is assigned a value of 1 if the data belongs to that month, and 0 otherwise. Sunchon City was selected as the control group because it is located in an inland area (not a border area), and it showed little change in lighting before and after border closure. As North Korea decided to close the border in January 2020, the event time point (reference month) was set at t = 4 in this study. ${\delta }_{t}$ is the time fixed effect that controls for common time trends. ${\theta }_{i}$ is the cell-fixed effect, which controls for time-invariant heterogeneity in light intensity within each cell. ${\varepsilon }_{i,g,t}$ is an error term. ${\beta }_{g,t}$ indicates differences in the logarithmic value of (light intensity + 1) in area $g$ relative to those in Sunchon City in month $t$ and compared with the difference between the two area groups in the reference month. The purpose of this study is to analyze changes in economic activity by observing the difference in night-time lights between cities in the border areas affected by the border closure policy and those that are not. It is difficult to directly prove a significant relationship between the total trade volume of North Korea and the night-time lights in border cities, and due to the limitation of the lack of additional data that can affect the night-time lights in border cities, this study could not include other explanatory variables. To overcome these limitations, this study compared the direction of changes in the flow of night-time lights with known changes in North Korea’s trade.

## Results

**Fig. 4:**
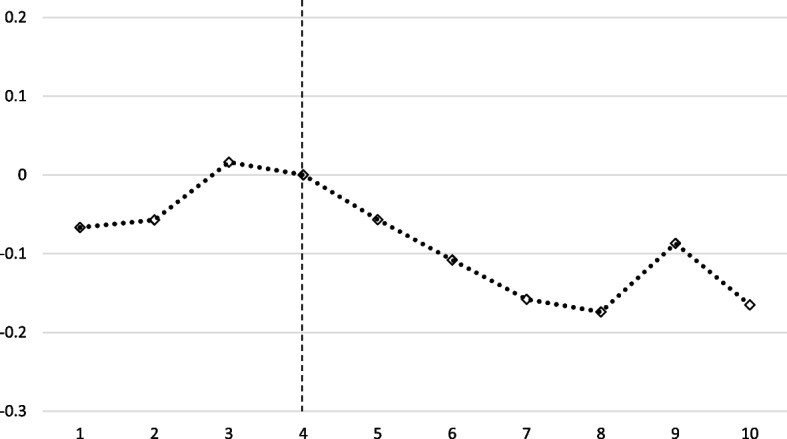
Night-time light response to the border closure. Note: The vertical dotted line at number 4 corresponds to January 2020, when border closures began. Each diamond mark represents a DID estimate. This study used the cluster standard error at the cell-level. Cell data for Pyongyang was not included in this regression analysis. The results of the DID estimates are in Table 3 in Appendix

Figure 4 shows the change of night-time light after border closure for all border trade cities. From January 2020 (number 4 in the graph), when the border blockade began, light steadily decreased. Light recovered slightly from May to June (numbers 8 and 9 in the graph); during this period, the number of COVID-19 confirmed cases in China decreased, and this suggests the possibility of a resumption of economic activities in the respective region.

**Fig. 5:**
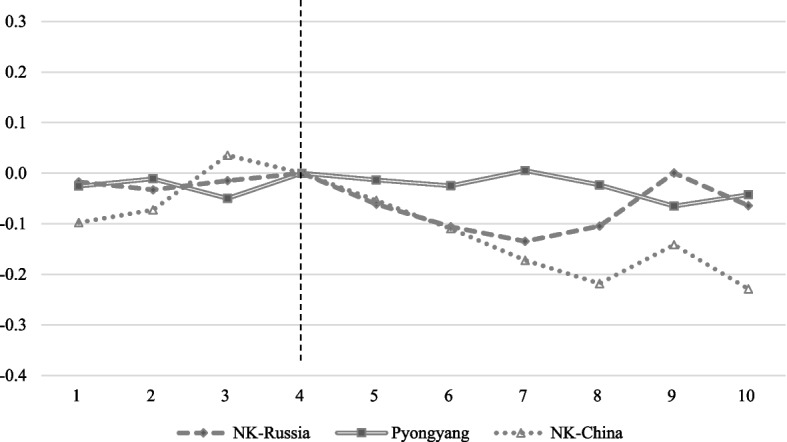
Night-time light response to the border closure by group. Note: The vertical dotted line at number 4 corresponds to January 2020, when border closures began. Each point represents a DID estimate. This study used the cluster standard error at the cell-level. The pre-trend coefficients of Chinese border cities were far from zero and gradually increased. They seemed to have increased as trade with China continued to increase from 2018 until the border closure owing to strengthened US sanctions against North Korea and China’s easing of trade with North Korea [41]. The DID estimates are in Table 4 in Appendix

Figure 5 shows the changes in night-time light after border closure in each group. In Pyongyang, which is not located in the border area, there was little change before and after the border blockade. When COVID-19 spread in China, North Korea claimed no confirmed cases. It is speculated that night-time light in Pyongyang, where major government agencies are located, did not change significantly because the border closure did not affect activities in Pyongyang. The impact of border closures seems to have been limited to border areas.

The change in lights in trading cities adjacent to the border between China and Russia decreased after the border closure. In the case of the Russian border area, light decreased until April (number 7 in the graph), after which it recovered. Conversely, in the case of the Chinese border area, the light decreased until May and seemed to recover again in June; however, unlike the Russian border area, the decreasing trend was maintained.

**Fig. 6:**
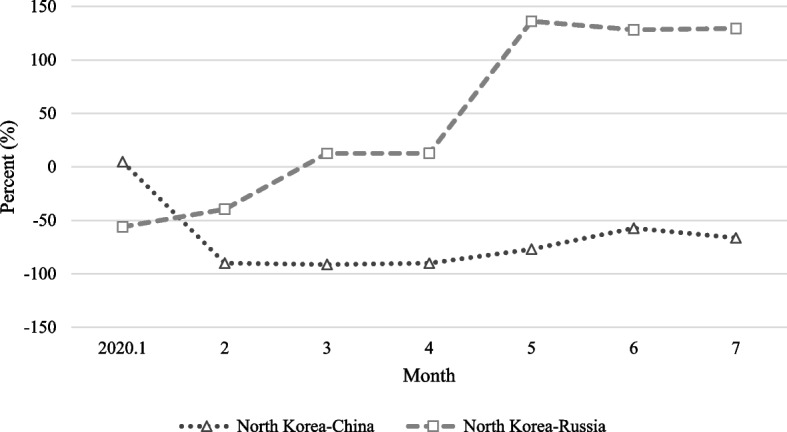
North Korea’s rate of change in trade compared to the same month of the previous year. Note: The information based on data provided by KITA

**Fig. 7:**
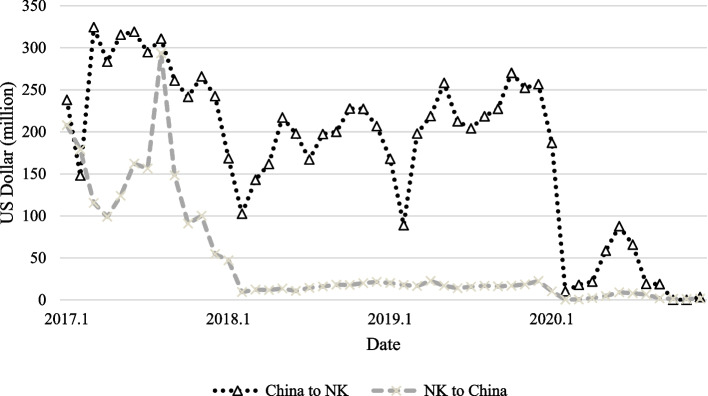
North Korea’s exports and imports to China. Note: The data was taken from the General Administration of Customs People’s Republic of China

Comparing the above results with trade data, it appears that the light change is related to North Korea’s trade activity. Figure 6 shows the rate of change in trade value between North Korea and China and between North Korea and Russia compared with the same month of the previous year. Since the border closure in January 2020, North Korea’s trade with China has declined considerably. Although exports had previously declined significantly because of the UN’s economic sanctions, imports had been maintained, as shown in Fig. 7. However, as border closures were implemented, imports were also greatly affected. On average, trade volume decreased by 78.5% from February to July compared with the same month of the previous year. This pattern is similar to the continuously dwindling lights in trade cities between North Korea and China.

In contrast, trade between North Korea and Russia showed a relatively rapid increase after the border closure. In January and February, the trade volume was low compared with the same months of the previous year; however, in March and April, it increased by approximately 12%. It can be seen that trade between North Korea and Russia has increased rapidly, with trade increasing by more than 100% from May compared with the same month of the previous year. From April, the intensity of light shows a pattern of recovery, and comparing this with trade data, it can be inferred that North Korea-Russia trade was actively carried out.

Since the outbreak of the COVID-19 pandemic, it is evident that there have been structural changes in North Korea’s imports. The imports from China to North Korea decreased by approximately 78%, from 2829.8 million dollars in 2019 to 601.7 million dollars in 2020, and exports also decreased from 261.1 million dollars to 52.1 million dollars [42]. All types of imports from China to North Korea decreased. In contrast, there was an increase in some types of imports from Russia. Table 2 shows the percentage change in North Korea’s imports from China and Russia. As food imports from China sharply decreased after the border closure, North Korea appears to have compensated by increasing imports from Russia. Moreover, there was a significant increase in medical imports, likely in preparation for the spread of the coronavirus.

**Table 2:** The percentage change in North Korea’s imports of food and medicine from 2019 to 2020

	Cereals,cereal preprtns	Fixed veg, Fats and oils	Medicinal, pharm	Sugars,molasses, honey
China	-74.4	-37.3	-55.8	-18.4
Russia	170.9	17.0	71.4	3000.0

This data is based on information publicly available on UN Comtrade Analytics, and has been modified by the author. All units are presented as percentages

Upon comparing changes in nighttime light and trade data, it seems that in the initial stages of the COVID-19 pandemic, North Korea made efforts to prevent the spread of the virus by decreasing trade activities with China and Russia. However, as the pandemic persisted, North Korea appeared to engage in trade activities through relatively stable routes, likely aiming to address the ongoing chronic food issues and improve the inadequate healthcare system.

## Discussion

This study analyzed changes in trade activities in North Korea’s trading cities after border closure using night-time light data and trade data. After the border closure was implemented in January 2020, the lights of trading cities adjacent to China and Russia continued to decline until April and May, when the number of COVID-19 confirmed cases in China began to decrease. Since then, the light intensity has recovered in Russian border trading cities; however, the light intensity in Chinese border cities has remained lower than pre-border closure. When compared with available trade data between North Korea and Russia and between North Korea and China, the change in light and trade volume show similar trends. According to the trade data, North Korea significantly reduced its trade volume with China in 2020 compared to 2019, while increasing its trade with Russia. The main imported goods from Russia were food and medicine.

The changes in nighttime light intensity in North Korea’s border areas could be interpreted in various ways. An increase in nighttime light may suggest heightened trade activities in the region, increased activation of hospital facilities due to a surge in patients in vulnerable border areas, and an escalation in military deployment to reinforce border closures. After the border closure, a shoot-to-kill order was issued for anyone approaching the border without permission, and North Korean residents face restrictions under the movement control order [43]. Hospitals, excluding those utilized by the elite or wealthy class, face challenges in electricity supply, making it practically difficult for North Korean residents to access healthcare [16]. Moreover, considering the economic situation in North Korea, a sudden increase in electricity supply and facility development for the sealed-off areas seems practically implausible. Taking these factors into account, changes in nighttime light intensity in border areas after the border closure are likely influenced by essential activities for the nation, such as material procurement.

In terms of changes in nighttime light and trade data, the result of this study highlights two implications regarding North Korea’s decision-making process between its economy and COVID-19 prevention during the pandemic. Firstly, North Korea prioritizes its epidemic prevention system. As China accounts for the majority of North Korea’s trade, with most of its major goods imported from China and exports directed to China, the decrease in trade with China could cause significant economic shocks to North Korea. Nonetheless, North Korea drastically reduced its trade with China through border closure. Moreover, despite not at the same level of trade with China, North Korea increased its trade with Russia for essential food and medicine, emphasizing the prioritization of epidemic prevention over its economic difficulties. This aspect is also evident in regional characteristics and transportation methods for materials. North Korea and China engage in trade via vehicles and trains crossing the Sino-Korean Friendship Bridge in Sinuiju, the Ji’An-Manpho Bridge in Manpho City, and the Ji’An Yalu River Border Railway Bridge, as well as other areas near the border. In terms of port routes, trade through shipping is feasible between the geographically proximate cities of Dandong and Sinuiju. In contrast, trade between North Korea and Russia is limited to train transportation through the Friendship Bridge located at the Tumen River in Rason City overland. In terms of port trade routes, the closest route for trade through shipping is the Rajin Port located in Rason City. Thus, it can be inferred that opting for trade with Russia through Rason City, which allows for minimal human contact by opening only the necessary areas while preventing the spread of the coronavirus, could have been the most optimal choice for importing food and medicine.

Secondly, the economic difficulties of North Korea are apparent. The study observed that North Korea actively expanded its trade with Russia, particularly in the food sector, following border closure. Prior to the COVID-19 pandemic, North Korea’s food supply was heavily reliant on imports from China, as domestic production alone could not meet the demand [16]. While food imports from China played a role in alleviating North Korea’s chronic food shortages, the border closure made it difficult to continue this reliance, prompting the need to explore alternative solutions. The restrictions on the procurement of goods due to the border closure, along with the control of movement between regions, would have rapidly decreased the supply of essential goods in the North Korean market, in addition to the economic sanctions imposed by the international community. The swift replacement of imports from China with imports from Russia in a short timeframe underscores the critical need for essential goods, emphasizing their importance. This implies that the longer the COVID-19 pandemic persists, the more challenging it would be for North Korea to maintain border closure due to economic difficulties.

## Conclusion

As the COVID-19 pandemic prolongs, there is a high possibility that North Korea would ease its border restrictions due to issues related to food shortages and inadequate medical supplies and facilities. Under the assumption of eased border restrictions, it is believed that the international community could provide support to North Korea. However, for the international support to effectively impact North Korea, three changes are deemed necessary. Firstly, cooperation from China and Russia is crucial. Before the COVID-19 pandemic, North Korea had a high dependency on trade with China and Russia and had severed exchanges with other countries. Consequently, North Korea might not actively seek direct assistance from the international community. Therefore, cooperation and exchanges between China, Russia, and the international community are essential. Secondly, a shift in perception regarding international medical supplies is needed within North Korea. The promotion of traditional medicine and discouragement of the use of foreign pharmaceuticals are prevalent within North Korea [16]. This suggests a likelihood of not utilizing supported medical supplies even if provided during the pandemic. Thirdly, addressing distribution issues is crucial. North Korea operates as a socialist state with a strong influence of the ruling class. Without clear distribution plans for food and medical supplies, there is a high probability of international assistance being monopolized by the privileged class and ruling elite, leading to imbalanced distribution. In conclusion, if these aspects do not undergo change, the international community’s assistance to North Korea during the COVID-19 pandemic may not be effective. Changes are needed both internally in North Korea and in the approach of the international community to understand the situation in North Korea and provide support accordingly for genuine international assistance to take place.

This study primarily examines the changes in North Korea’s economic activities during the early stages of the COVID-19 pandemic, with a focus on the border areas. Observing North Korea’s initial response to the COVID-19 pandemic can provide insights and predictions for future actions. However, to comprehensively understand the long-term changes in North Korea, further research specifically addressing this aspect is deemed necessary. Furthermore, to comprehend the changes in North Korea, there is a need for continuous analysis of the behavioral changes of China and Russia towards North Korea.

The limitation of this study is associated with the representativeness of the data. Although many studies use nighttime light data to estimate changes in economic activity, it is important to recognize that nighttime light alone cannot entirely substitute for economic activities. This limitation stems from the fact that nighttime light, being an inherent characteristic of the data, is not an economic variable. While it can be employed as an alternative data source to infer economic activities, it lacks the comprehensive nature necessary for fully representing such activities. Despite attempts in this study to address this limitation through comparisons with trade data, the inherent constraints of the data persist. Therefore, it is acknowledged that future research on the utilization of nighttime light should thoughtfully consider these limitations and explore additional data sources that can complement these drawbacks.

## Data Availability

The night-time light data supporting this study’s findings are available from the following link: https://eogdata.mines.edu/products/vnl/. Data on the number of confirmed cases in China were obtained from the WHO, and trade data were obtained from the United Nations Comtrade and General Administration of Customs People’s Republic of China (http://english.customs.gov.cn/statics/report/monthly.html).

## Appendix

**Table 3:** Results of DID estimates of trade cities located in border areas

	**Light**
**NK trade cities**	
X Month -3	-0.067***
	(0.015)
X Month -2	-0.057***
	(0.011)
X Month -1	0.016
	(0.010)
X Month + 1	-0.057***
	(0.009)
X Month + 2	-0.108***
	(0.008)
X Month + 3	-0.158***
	(0.009)
X Month + 4	-0.174***
	(0.011)
X Month + 5	-0.087***
	(0.011)
X Month + 6	-0.165***
	(0.012)
Constant	0.626***
	(0.003)
Observations	29,030
R-squared	0.919

The parentheses indicate cluster standard errors at the cell level. *** *p* < 0.01. The analysis in this study incorporates cell- and time-fixed effects

**Table 4:** Results of DID estimates for each trade city group located in the border area

	**Light**
**NK-China trade cities**	
X Month -3	-0.098***(0.016)
X Month -2	-0.072***(0.012)
X Month -1	0.036***(0.011)
X Month + 1	-0.054***(0.009)
X Month + 2	-0.110***(0.008)
X Month + 3	-0.172***(0.010)
X Month + 4	-0.218***(0.012)
X Month + 5	-0.142***(0.012)
X Month + 6	-0.229***(0.013)
**Pyongyang**	
X Month -3	-0.026*(0.015)
X Month -2	-0.011(0.010)
X Month -1	-0.050***(0.010)
X Month + 1	-0.013(0.009)
X Month + 2	-0.025***(0.007)
X Month + 3	0.005(0.008)
X Month + 4	-0.023**(0.010)
X Month + 5	-0.065***(0.011)
X Month + 6	-0.042***(0.011)
**NK-Russia trade cities**	
X Month -3	-0.017(0.016)
X Month -2	-0.033***(0.012)
X Month -1	-0.015(0.012)
X Month + 1	-0.061***(0.011)
X Month + 2	-0.106***(0.010)
X Month + 3	-0.135***(0.011)
X Month + 4	-0.105***(0.012)
X Month + 5	0.000(0.013)
X Month + 6	-0.064***(0.014)
Constant	0.675***(0.002)
Observations	53,970
R-squared	0.942

The parentheses indicate cluster standard errors at the cell level. *** *p* < 0.01, ** *p* < 0.05, **p* < 0.1. The analysis in this study incorporates cell- and time-fixed effects

## Abbreviations

DID: Difference in difference
NTL: Night-time light
EOG: Earth Observation group
KITA: Korea international trade association
UNOCHA: United Nations Office for the Coordination of Humanitarian Affairs
